# Patterns of risk—Using machine learning and structural neuroimaging to identify pedophilic offenders

**DOI:** 10.3389/fpsyt.2023.1001085

**Published:** 2023-04-20

**Authors:** David Popovic, Maximilian Wertz, Carolin Geisler, Joern Kaufmann, Markku Lähteenvuo, Johannes Lieslehto, Joachim Witzel, Bernhard Bogerts, Martin Walter, Peter Falkai, Nikolaos Koutsouleris, Kolja Schiltz

**Affiliations:** ^1^Department of Psychiatry and Psychotherapy, Ludwig-Maximilians-University Munich, Munich, Germany; ^2^Department of Forensic Psychiatry, Ludwig-Maximilians-University Munich, Munich, Germany; ^3^International Max Planck Research School for Translational Psychiatry (IMPRS-TP), Munich, Germany; ^4^Max Planck Institute of Psychiatry, Munich, Germany; ^5^Department of Dermatology, Venereology, and Allergology, Charité - Universitätsmedizin Berlin, Berlin, Germany; ^6^Department of Neurology, Otto-von-Guericke-University, Magdeburg, Germany; ^7^Department of Forensic Psychiatry, University of Eastern Finland, Niuvanniemi Hospital, Kuopio, Finland; ^8^Institute for Molecular Medicine FIMM, University of Helsinki, Helsinki, Finland; ^9^Central State Forensic Psychiatric Hospital of Saxony-Anhalt, Uchtspringe, Germany; ^10^Salus Institut, Salus gGmbH, Magdeburg, Germany; ^11^Department of Psychiatry and Psychotherapy, Otto-von-Guericke-University, Magdeburg, Germany; ^12^Department of Psychiatry and Psychotherapy, Jena University Hospital, Jena, Germany; ^13^Institute of Psychiatry, Psychology and Neuroscience, King’s College London, London, United Kingdom

**Keywords:** pedophilia, MRI, child sexual abuse (CSA), support vector machines, machine learning, forensic psychiatry

## Abstract

**Background:**

Child sexual abuse (CSA) has become a focal point for lawmakers, law enforcement, and mental health professionals. With high prevalence rates around the world and far-reaching, often chronic, individual, and societal implications, CSA and its leading risk factor, pedophilia, have been well investigated. This has led to a wide range of clinical tools and actuarial instruments for diagnosis and risk assessment regarding CSA. However, the neurobiological underpinnings of pedosexual behavior, specifically regarding hands-on pedophilic offenders (PO), remain elusive. Such biomarkers for PO individuals could potentially improve the early detection of high-risk PO individuals and enhance efforts to prevent future CSA.

**Aim:**

To use machine learning and MRI data to identify PO individuals.

**Methods:**

From a single-center male cohort of 14 PO individuals and 15 matched healthy control (HC) individuals, we acquired diffusion tensor imaging data (anisotropy, diffusivity, and fiber tracking) in literature-based regions of interest (prefrontal cortex, anterior cingulate cortex, amygdala, and corpus callosum). We trained a linear support vector machine to discriminate between PO and HC individuals using these WM microstructure data. *Post hoc*, we investigated the PO model decision scores with respect to sociodemographic (age, education, and IQ) and forensic characteristics (psychopathy, sexual deviance, and future risk of sexual violence) in the PO subpopulation. We assessed model specificity in an external cohort of 53 HC individuals.

**Results:**

The classifier discriminated PO from HC individuals with a balanced accuracy of 75.5% (sensitivity = 64.3%, specificity = 86.7%, *P*_5000_ = 0.018) and an out-of-sample specificity to correctly identify HC individuals of 94.3%. The predictive brain pattern contained bilateral fractional anisotropy in the anterior cingulate cortex, diffusivity in the left amygdala, and structural prefrontal cortex-amygdala connectivity in both hemispheres. This brain pattern was associated with the number of previous child victims, the current stance on sexuality, and the professionally assessed risk of future sexual violent reoffending.

**Conclusion:**

Aberrant white matter microstructure in the prefronto-temporo-limbic circuit could be a potential neurobiological correlate for PO individuals at high-risk of reoffending with CSA. Although preliminary and exploratory at this point, our findings highlight the general potential of MRI-based biomarkers and particularly WM microstructure patterns for future CSA risk assessment and preventive efforts.

## Introduction

Child sexual abuse (CSA) is defined as any completed or attempted sexual act or contact with a child, as well as any form of sexual exploitation of a child (1). CSA is a universal problem with global prevalence rates of 20% for girls and 8% for boys (2). Along with its profound short- and long-term consequences, CSA poses a major challenge to society (3). Personal consequences for the victim range from physical effects (e.g., injury, chronic pain, sexual dysfunction) and psychiatric disorders (e.g., depression, post-traumatic stress disorder, substance abuse) to emotional and interpersonal issues (4). CSA also amounts to a substantial economic burden, as the annual cost of CSA is estimated at $ 9.34 billion in the US alone (5). Therefore, lawmakers and public health officials are moving toward preventive measures as opposed to the current reactive paradigm, which requires waiting until a child has been victimized. Effective prevention initiatives reduce not only the number of children sexually victimized, but also the costs associated with CSA (3). While primary prevention of CSA involves wide-scale initiatives aimed at the general public (e.g., general crime deterrence, public education, adequate sex education in schools), secondary and tertiary prevention focuses on decreasing the risk of offending or preventing recidivism among those who are at risk of engaging in or have already committed CSA (6).

To effectively allocate preventive resources, it is crucial to identify and stratify individuals who belong to pedophilic offending risk groups. Pedophilic offending includes hands-on crimes, that is, actual sexual contact with a child, as well as hands-off crimes, namely the use of media depicting CSA (7–9). In the context of this study, the term “pedophilic offenders” (PO) is used exclusively to refer to hands-on offenders who commit CSA. Although CSA is a multifactorial process, pedophilia, the primary, or exclusive sexual attraction to prepubescent children, is one of the main risk factors for CSA (10). Pedophilia is present in approximately 50% of convicted child sexual offenders, but not all pedophilic individuals commit CSA (11–13). Therefore, several clinical and actuarial tools have been developed to assess the potential risk of sexual reoffending (14). However, validated tools specifically designed for child sexual reoffending are not yet available. Furthermore, these risk assessment tools are mostly applicable to individuals who have already been convicted of a violent and/or sexual crime. For individuals without a prior conviction, there are no validated measures of this type, specifically not for CSA (15). This becomes particularly troubling since up to 95% of the individuals prosecuted for CSA are first-time offenders (3). Another sign that current instruments could still be improved is the fact that only 10–50% of CSA cases are prosecuted, while that likelihood decreases even further when the victim is a preschooler (16). Furthermore, a meta-analysis of the most established actuarial and structured professional judgment (SPJ) tools (SORAG, Static-99, SVR-20) reported a balanced accuracy of only 61%, defined as the mean of sensitivity and specificity, in a pooled sample of 20 study cohorts, containing approximately 10,000 individuals from multiple countries (17). A key limitation of these tools could be that they are based solely on clinical and/or sociodemographic data (14, 17, 18). Like other fields of psychiatry, discovering predictive neurobiological signatures and therefore possibly generating biomarkers for PO individuals could tap into a new level of information on these individuals and therefore, in connection with already established tools, help to better identify those pedophilic individuals who are at risk of committing CSA (10, 14, 17). So far, structural neuroimaging studies were unable to robustly identify gray matter volume (GMV) patterns associated with the diagnosis of pedophilia (19). The GMV patterns found in previous neuroimaging studies, specifically in orbitofrontal, limbic, and basal ganglia structures, turned out to be much more closely associated with PO individuals than with pedophilia alone (19–23). Consequently, diffusion-tensor imaging (DTI) studies reported an increase in fractional anisotropy (FA) in the white matter of the left hemisphere (24) and FA reductions in the corpus callosum (23) in PO individuals compared to healthy control subjects and non-offending pedophilic individuals. Therefore, it has been suggested that PO individuals could have the most distinct neurobiological footprint of all these groups (10). Due to their commission of pedosexual crimes, the PO subpopulation is also forensically the most relevant subpopulation (10, 20, 21).

For that reason, we decided to explore brain structural correlates of PO individuals using advanced machine learning techniques, which have proven to be highly valuable in tackling the biological elusiveness of complex disorders and phenotypes (25). We present an exploratory proof-of-principle study that uses supervised machine learning and structural magnetic resonance imaging to derive a neuroanatomical model for PO individuals and evaluate its clinical utility in diagnosis and risk assessment. In a sample of 14 PO and 15 healthy control (HC) individuals, we analyzed DTI data from a literature-based selection of brain regions (prefrontal cortex, anterior cingulate cortex, amygdala, and corpus callosum) (20, 21), trained a support vector machine on these MRI features to distinguish between PO and HC individuals and applied the final PO model to an external sample of 53 HC individuals for further evaluation of model specificity. In addition, we used stepwise linear regression to investigate the clinical and forensic implications of the PO model with respect to measures of psychopathy, delinquency, sexual deviance, and sociodemographic characteristics. In this pilot study, our aim was to demonstrate that the combination of neuroimaging and machine learning provides the opportunity to discover a predictive brain signature for PO individuals. This approach could serve as a template for future MRI biomarker studies in the context of secondary and tertiary prevention of CSA.

## Materials and methods

### Study participants

The main study sample was used for the generation of the PO model and an external HC sample to evaluate out-of-sample specificity. The main study sample, which was previously reported by Schiltz et al. (26), contained 14 male PO individuals (unmedicated forensic inpatients, in part under supportive psychotherapy), who had committed sexual offenses involving children younger than 10 years of age and met the diagnostic criteria for pedophilia according to the DSM-IV-R following the Structured Clinical Interview for DSM-IV (SCID-IV) (27). At the time of the study, they were inpatients at the Forensic Psychiatric State Hospital in Uchtspringe, Saxony-Anhalt, Germany. The main study sample also contained 15 age and education-matched HC individuals who did not meet the criteria for any psychiatric disorder according to SCID-IV. The external HC sample consisted of 53 non-medicated HC individuals (age, *μ* = 23.77, SD = 3.02), which were scanned in the same MRI unit as part of a different study cohort (28). The HC individuals in the main study sample and in the external HC sample did not receive further forensic evaluation. Exclusion criteria for both samples were presence of other psychiatric or neurological disorders, alcohol or drug abuse, head trauma, antiandrogen medication, and general MRI study exclusion criteria (i.e., claustrophobia, metal implants). The authors assert that all procedures contributing to this work comply with the ethical standards of the relevant national and institutional committees on human experimentation and with the Declaration of Helsinki of 1975, as revised in 2008 (29). All procedures involving human subjects/patients were approved by the local ethics advisory board of the medical school of Otto-von-Guericke University, Magdeburg. Written informed consent was obtained from all study participants.

### Sociodemographic and forensic assessment

All study participants were rated by experienced forensic psychiatrists. Verbal premorbid intelligence was measured using the German Vocabulary Test (“Wortschatz–Intelligenztest”) (30). A specific forensic evaluation was performed for all PO individuals. The Sexual Violence Risk-20 checklist (SVR-20) is a structured professional judgment tool (SPJ), which operationalizes the risk of reoffending with a sexual violent crime in three domains (“psychosocial adjustment,” “history of sexual offenses,” and “future plans”). It also contains a final three-level assessment of risk for future sexual violent reoffences ranging from “low” (0) and “moderate” (1) to “high” (2), which yielded high predictive validity in previous studies (31). The Psychopathy Checklist-Revised by Hare (PCL-R) is another SPJ tool, which covers two factors (“interpersonal” and “social deviance”) and four facets (“interpersonal,” “affective,” “lifestyle,”and “antisocial”) of the concept of psychopathy (32). SVR-20 and PCL-R are known to reliably reflect features relevant to criminological characterization and risk assessment (33). The Multiphasic Sex Inventory (MSI) is a self-report measure that assesses psychosexual characteristics in adult male sexual abusers and rapists in four domains: “course and behavior patterns of sexual deviance” (subdivided into “child sexual abuse,” “rape,” and “exhibitionism”), “paraphilias,” “sexual dysfunctions,” and “sexual knowledge and beliefs” [(34), see Supplementary Table 1].

### MRI data acquisition and pre-processing

MRI data from the main study sample and the external HC sample were acquired on a General Electric Signa LX 1.5-T scanner located at the University of Magdeburg, Germany. Data for diffusion tensor calculations were collected with 12 non-collinear gradient orientations, each additionally measured with the opposite diffusion gradient polarity (35). The orientations were chosen according to the DTI acquisition scheme proposed by Papadakis et al. (36). The total of 24 diffusion-weighted measurements, each an average of four measurements, was divided into four blocks, each preceded by a non-diffusion-weighted acquisition. The DTI images were eddy-current corrected according to the correction scheme of Bodammer et al. (35) followed by a correction of head movement based on the non-diffusion-weighted images using the AIR software package (37). The diffusion tensors were calculated for each voxel and further decomposed into eigenvalues and eigenvectors. Based on the eigenvalues, the mean diffusivity (MD) and fractional anisotropy (FA) as well as the axial diffusivity (AD) and radial diffusivity (RD) coefficients were determined. FA, MD, AD, and RD values of HC and PO individuals were read from a pre-defined set of regions of interest (ROIs), which have previously been implicated in sexual arousal and/or pedophilia (20, 38–41). The Matlab-based MarsBaR ROI toolbox (version 0.44) was used to create spherical ROIs of either 5 mm (ACC) or 7 mm (amygdala) radius with a 1 mm resolved MNI template as the underlying image. DTI values were taken from 10 different ACC regions (5 each on the right and left) as suggested by Kelly et al. (42) and 2 amygdala regions with MNI coordinates taken from the work of Motomura et al. (43) (see Supplementary Table 2 for MNI coordinates). Additionally, the corpus callosum (CC) was parcellated into five different segments according to the Hofer-Frahm scheme (44). The selected CC ROIs had the following volumes from CC1 to CC5 (anterior to posterior): 2.365, 2.373, 0.838, 0.395, and 2.941 cm^3^. The sum of all five ROIs (total CC) amounts to 8.912 cm^3^. The CC ROIs were located midsagittally and had a left-right extension of 11 mm (from −5 to +5 mm).

Fiber tract reconstruction was carried out using a previously described double-step probabilistic approach (45). As seed and target regions, automatically segmented amygdala and prefrontal cortex (PFC) regions based on T1-weighted volumes and generated by the freesurfer recon-all pipeline [freesurfer version 6.0.0 (46)] were used.^1^ The regions were co-registered with the diffusion data using the FSL tools flirt and fnirt (47) and fiber tracking was performed in the diffusion space. Seed and target regions were individually different in size between 2.6 and 3.8 cm^3^ for the amygdala and between 59 and 101 cm^3^ for the PFC regions. Normalized fiber counts were used as an estimate of structural connectivity (SC). Due to this approach, the amygdala region used for fiber tracking slightly differed from the one used for acquisition of the DTI anisotropy and diffusivity parameters. This approach also led to individual, non-spherical amygdala and PFC regions, for which MNI coordinate description is not suggested. A total of 68 anisotropy/diffusivity features (17 features each for FA, MD, RD, AD) and 4 SC features (prefrontal cortex-amygdala/amygdala-prefrontal cortex left/right) entered the analysis (see also Supplementary material—MRI data acquisition).

### Machine learning pipeline

Using the machine learning software NeuroMiner, version 1.05^2^ (48), we built a support vector machine (SVM) classifier with a linear kernel to discriminate between PO and HC individuals. The SVM algorithm is known to be robust when performing prediction analyses at low sample sizes (49). We embedded the algorithm in a repeated nested cross-validation (CV) framework using 10-folds and 10 permutations each in the inner (CV1) and outer (CV2) cycle to prevent overfitting and increase generalizability (Supplementary material—Machine learning pipeline) (25). The CV1 cycle was used for hyperparameter optimization. The best model from the CV1 level was applied to the outer CV2 fold, which contained individuals who had not been used to train the model. Furthermore, we used a wrapper-based ensemble generation strategy (greedy forward feature selection) at the CV1 level, which generated a final parsimonious model that only contained the most relevant subset of predictive features (50). The significance of the final model was assessed by comparing the performance of the final model against a null distribution of 5,000 models trained on random permutations of the target labels (Supplementary material—Permutation testing) (51). To assess out-of-sample specificity of the PO model, we applied it to an external sample of 53 HC individuals.

### Model performance evaluation

The performance of the final model was evaluated with different performance metrics. The main performance metric, which was also the optimization criterion for model training, was the measure of balanced accuracy (BAC), defined as the mean of sensitivity and specificity. Furthermore, we used the sensitivity and specificity of the model to calculate the number needed to diagnose (NND), which is the inverse of Youden’s index given by [1/(sensitivity + specificity – 1)]. The NND displays how many individuals have to be examined in order to correctly detect one individual with the phenotype of interest (52). However, this measure assumes that the study population, in which the model was generated, is representative of the distribution of the phenotype in real-world populations. Since the prevalence of PO individuals in our study sample differs considerably from the prevalence of PO individuals in the general population as well as in other real-world cohorts (e.g., psychiatric and forensic inpatient settings, specialized outpatient services) additional metrics such as the predictive summary index (PSI) and its reciprocal, the number needed to predict (NNP), were used. The PSI and the NNP allow to factor in the actual prevalence of a certain phenotype and thus reflect the performance of the model in possible real-world scenarios (52). The PSI is a combination of PPV and NPV given by (PPV + NPV – 1). Through PPV and NPV, the performance of the model can be estimated using varying prevalence rates, which impact both the PPV and the NPV (53). The PSI reflects the additional overall certainty of performing a certain test based on knowledge of the prevalence of the phenotype. The NNP, the inverse of the PSI, is the number of people who need to be examined by the test or the model to correctly predict one individual with the phenotype of interest in a population with a given prevalence of the phenotype of interest (52, 53).

### Model visualization

In our case, a binary classification (−1 vs. 1) was carried out, whereas PO individuals were defined as positive label (numerical: 1), i.e., the phenotype of interest, while HC individuals were defined as negative label (numerical: −1). SVM models place weights on all input features, ranging from −1 to 1. SVM models then predict the labels by multiplying an individual’s feature values (i.e., anisotropy, diffusivity, or connectivity values in the 72 input features) with the model feature weights. In our case this leads to 72 multiplication products which are then aggregated into a final decision score. If the decision score is positive, then the positive label (in this case: PO) is predicted, while a negative decision score leads to the prediction of the negative label (in this case: HC). Before entering the analysis pipeline, all input features are rescaled from 0 to 1, so that beyond zero only positive features enter the analysis. Therefore, multiplication of a positive feature weight with the corresponding feature value of an individual always yields a positive multiplication product, while a negative feature weight always yields a negative multiplication product. Consequently, a positive feature weight indicates that the corresponding feature increases the decision score and therefore pushes the model toward prediction of the positive label, while a negative feature weight decreases the decision score and pushes the model toward prediction of the negative label. The absolute value of the feature weight further indicates how strongly a certain feature contributes to either a positive or a negative decision score. Finally, the distance between the decision score and the decision boundary (i.e., the zero value) reflects the prediction certainty of the model. Higher positive decision scores or lower negative decision scores reflect that the model is more certain of either the positive or negative label prediction. The closer the decision score gets to zero, the more uncertain a model is of its prediction. If a feature weight is zero, the corresponding feature does not influence the decision score and therefore also does not impact the prediction, regardless of the input feature value of the individual.

To obtain a stable visualization of such an SVM model and its feature weights, we used measures of pattern element stability (cross-validation ratio) and pattern element significance (sign-based consistency) (54). The CVR as a measure for pattern stability was inspired by the bootstrap ratio commonly used in the Partial Least Squares literature and described by Krishnan et al. (55). The CVR of a feature weight aggregates the feature weights of a certain feature across all models computed in the CV structure. The CVR is therefore similar to a Z score of the feature weight and provides a more stable estimate of how a certain feature was weighted in all computed models. In our case, the 10-folds and 10 permutations on the CV1 and CV2 level leads to the computation of 10,000 models, thereby making the CVR particularly stable. The CVRs of the feature weights are interpreted in the same way as the feature weights. Positive CVRs indicate that a certain feature was on average positively weighted and contributed to the prediction of the positive label, while negative CVRs indicate the opposite. Similar to the feature weights, the absolute values of the CVRs indicate how strongly a feature influenced the decision toward the positive or the negative label. Higher positive CVRs or lower negative CVRs reflect that the corresponding feature pushed the overall decision more strongly toward the positive or the negative label. In the current analysis, positive CVRs indicate that a certain feature was, on average, predictive of PO individuals, while negative CVRs indicate that the feature was, on average, predictive of HC individuals.

Complementary to the CVR measure, we implemented a sign-based consistency method, which is based on an approach proposed by Gómez-Verdejo et al. (56) toward wrapper-based feature selection strategies. The sign-based consistency method assesses how consistently a feature was weighted positively or negatively across all computed models. Using that method, a *P*-value can be computed for each feature, determining whether the rate at which that feature received either a positive or negative sign (i.e., PO vs. HC prediction) exceeded chance level. Therefore, this method quantifies how consistently a feature contributed to the prediction of a certain target and whether that rate is significant beyond chance level.

### Univariate analysis

Group-level differences were assessed using the non-parametric Mann–Whitney-U-Test. *Post-hoc* investigation of the PO model was performed using all available sociodemographic data (age, verbal intelligence, years of education) and forensic information (number of child victims, total and subscale scores of SVR-20, PCL-R, and MSI as seen in Table 1) to predict SVM classifier scores via stepwise linear regression analysis (stepwiselm function, Matlab R2020b). All analyses were false discovery rate-corrected (FDR) (57) for multiple tests at a significance threshold of *q* = 0.05.

**TABLE 1:** Clinical and sociodemographic characteristics of the study sample.

	PO	HC	χ ^2^/U^a^	*P*
Sample size	14	15		
Age, years	40.07 (8.76)	44.13 (11.53)	81.00	0.31
Verbal intelligence^b^	106.50 (10.72)			
Years of education	12.50 (1.45)	12.71 (0.52)	92.50	0.59
Right-handedness	13 (92.9%)	14 (93.3%)	0.0026	0.96
Number of child victims	4.50 (2.81)			
**Sex of victims**				
Exclusively male/female	3/6			
Male and female	5			
**SVR-20**				
Final assessment score	1.57 (0.65)			
Psychological adjustment	6.86 (2.95)			
Sexual offenses	4.93 (2.15)			
Future plans	1.00 (1.07)			
Total score	12.79 (5.20)			
**PCL-R**				
Interpersonal traits: Factor 1	5.00 (4.26)			
Social deviance: Factor 2	3.79 (3.71)			
Total score	9.07 (8.06)			
**MSI**				
Sexual abuse of children	21.93 (5.69)^c^			
Rape	3.79 (6.17)			
Exhibitionism	1.79 (1.26)			
Paraphilias	3.50 (3.64)^c^			
Sexual dysfunctions	4.93 (2.99)			
Sexual knowledge and beliefs	15.93 (3.67)			

Results are stated as mean value followed by its standard deviation in brackets: μ (SD). PO, pedophilic offenders; HC, healthy control individuals; *P*, *P*-value; SVR-20, Sexual Violence Risk-20; PCL-R, Psychopathy Checklist-Revised; MSI, Multiphasic Sex Inventory. The *P*-values are stated after false discovery rate correction for multiple testing (the table represents a family of tests).

^a^Mann–Whitney U test (U test).

^b^Measured via the German Vocabulary Test [“Wortschatz–Intelligenztest” (30)].

^c^Above average results in comparison to a German norm sample of child abuser (*n* = 230) (34).

## Results

### Group-level characteristics

In the PO population, the number of previous child victims ranged from 1 to 10 (Table 1). Six PO individuals had committed CSA exclusively against girls, three exclusively against boys, and the remaining five had committed CSA involving both girls and boys. No significant differences were detected between the HC and PO individuals with respect to age (*U* = 81.00; *P* = 0.31) and years of education (*U* = 92.50; *P* = 0.59). Compared to a German norm sample of 230 PO individuals (34), the study PO individuals showed elevated scores in the MSI domain “course and behavior patterns of sexual deviation” (Table 1). Specifically, they showed significantly higher scores on the subscale “child sexual abuse” (*μ* = 21.93; SD = 5.69) and its subdomains “fantasy” (*μ* = 6.07; SD = 2.28), “searching/sneaking around and persuasion tactics” (*μ* = 5.43; SD = 2.26) and “sexual assault/attack” (*μ* = 6.71; SD = 1.48). Furthermore, elevated levels were found in the “paraphilia” domain (*μ* = 3.50; SD = 3.64), which were driven by significantly higher scores in the “voyeurism” sub-domain (*μ* = 1.57; SD = 1.64). PCL-R scores of PO individuals (*μ* = 9.07; SD = 8.06) were below the 95% confidence interval of non-standardized samples of incarcerated violent offenders (58), forensic inpatients (59), and PO individuals released from prison (60). SVR-20 total scores of the PO individuals (*μ* = 12.79; SD = 5.20) were below the 95% confidence interval of non-standardized samples of incarcerated violent and sexual offenders (61, 62). Furthermore, group-level testing of all 72 input features (68 anisotropy/diffusivity features and 4 SC features) did not yield any significant differences between HC and PO individuals (Supplementary Table 3).

### Cross-validation and out-of-sample specificity validation results

The classifier correctly identified 9 of 14 PO individuals (sensitivity = 64.3%) and 13 of 15 HC individuals (specificity = 86.7%), with a cross-validated BAC of 75.5%, a positive predictive value (PPV) of 82.0% and a negative predictive value (NPV) of 72.0% (Table 2). The PO model also yielded a number needed to diagnose (NND) of 1.96 and an area under the curve (AUC) of 0.69. The PO model was significant against 5,000 permutations of the target labels (*P*_5000_ = 0.018). When applied to the external HC sample, the PO model correctly identified 50 of 53 as HC individuals (specificity = 94.3%).

**TABLE 2:** Prediction performance in the main study and external HC sample.

	TP	TN	FP	FN	Sens (%)	Spec (%)	BAC (%)	PPV (%)	NPV (%)	NND	PSI	AUC	*P*_5000_
**Main study sample**													
PO vs. HC	9	13	2	5	64.3	86.7	75.0	82.0	72.0	1.96	0.540	0.69	0.018
**External HC sample**													
HC	0	50	3	0	–	94.3%	–	0.0	100.0	–	–	–	–

TP, number of true positives; TN, number of true negatives; FP, number of false positives; FN, number of false negatives; Sens, sensitivity; Spec, specificity; BAC, balanced accuracy; PPV, positive predictive value; NPV, negative predictive value; NND, number needed to diagnose; AUC, area-under-the curve. *P*_5000_, *P*-value calculated by comparing the BAC of the final PO model against a null distribution of 5,000 models trained on random permutations of the target labels.

When given assumed prevalence rates of PO individuals between 1 and 75%, the model yielded robust PPV, NPV, PSI, and NNP values (Table 3). Specifically, the model produced PSI values greater than 40% and an NNP lower than 3 for assumed prevalence rates ranging from 20 to 70%, peaking with a PSI of 55% and an NNP of 1.83 at a prevalence rate of 40%.

**TABLE 3:** Advanced performance metrics of the PO model at varying prevalence rates of PO individuals.

Prevalence (%)	PPV (%)	NPV (%)	PSI (%)	NNP
1	5	100	4	23.57
5	20	98	18	5.51
10	35	96	31	3.27
20	55	91	45	2.20
30	67	85	52	1.91
40	76	78	55	1.83
50	83	71	54	1.86
60	88	62	50	2.01
70	92	51	43	2.33

PPV, positive predictive value; NPV, negative predictive value; PSI, predictive summary index; NNP, number needed to predict. Depicted are the performance metrics of the PO model under the assumption of different prevalence rates of PO individuals ranging from 1 to 70%.

### Model investigation

**FIGURE 1:**
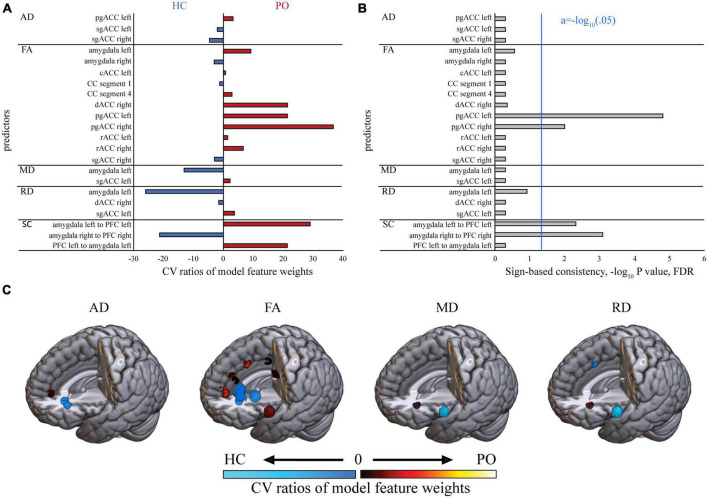
Discriminative brain pattern of the PO model. **(A)** Depicted are the CV ratios (CVRs) of the feature weights of the PO model, sorted by DTI modality. Positive CVRs, predictive of PO individuals, are displayed in red, and negative CVRs, predictive of HC, are displayed in blue. **(B)** Depicted are the *P*-values, computed via sign-based consistency mapping. *P*-values surpassing the FDR-adjusted threshold indicate which features were consistently weighted in the same direction (positively or negatively) exceeding chance level across all computed CV models. **(C)** The open-source 3-dimensional rendering software MRIcroGL (McCausland Center for Brain Imaging, University of South Carolina; https://www.nitrc.org/projects/mricrogl/) was used to overlay the CVR maps on the Montreal Neurological Institute single-participant template and produce 3-dimensional renderings. The cool color scale indicates lower (i.e., predictive of HC individuals) and the warm color scale higher (i.e., predictive of PO individuals) CVRs. SC, structural connectivity; FA, fractional anisotropy; RD, radial diffusivity; AD, axial diffusivity; MD, mean diffusivity; HC, healthy control individuals; PO, pedophilic offender individuals; cACC, caudal anterior cingulate cortex; dACC, dorsal anterior cingulate cortex; rACC, rostral anterior cingulate cortex; sgACC, subgenual anterior cingulate cortex; pgACC, pregenual anterior cingulate cortex; CC, corpus callosum; PFC, prefrontal cortex; CVR, cross validation ratio.

Using a wrapper-based feature selection, 22 of 72 input features were included in the final PO model (Figure 1). The CV ratios (CVRs) of the feature weights, illustrating how predictive a feature was for HC (CVR < 0) or PO (CVR > 0) across all computed CV models, produced a distinct white matter (WM) microstructure pattern (Figures 1A, C). Higher FA in the left amygdala (CVR = 9.19), the right dACC (CVR = 21.45), bilaterally in the pgACC (right: CVR = 36.76, left: CVR = 21.42) and in the right rACC (CVR = 6.65) was strongly predictive of PO individuals. Smaller, negative CVRs of FA feature weights, indicative of HC individuals, were found in the right amygdala (CVR = −3.10), the CC segment 1 (CVR = −1.24), and the right sgACC (CVR = −3.01). The RD domain yielded 3 features, of which higher RD in the left amygdala strongly predicted HC individuals (CVR = −26.03). The MD domain was represented with two features, of which higher MD in the left amygdala was highly predictive of HC individuals (CVR = −13.16). The model also contained 3 weaker AD features (pgACC left, sgACC left/right), which received CVRs between −4.66 and 3.24. Furthermore, stronger SC values in the left hemisphere were among the main predictors of PO individuals (left amygdala to left PFC: CVR = 29.05, left PFC to left amygdala: CVR = 21.41), while higher signaling in the right hemisphere (right amygdala to right PFC: CVR = −21.35) was shown to be highly predictive of HC individuals.

Sign-based consistency analysis determined four features to be the most reliable predictors: FA pgACC left/right and SC amygdala to PFC left/right (Figure 1B).

### *Post-hoc* assessment of possible confounders and forensic utility

Given the complete set of sociodemographic and forensic information on PO individuals, the stepwise linear regression analysis successfully predicted the brain-based PO model decision scores (Table 4, *F* = 26.1, *P* = 5.75E-05, df = 9). The prediction was based only on three features: The final SVR-20 assessment score (estimate = 3.28, 95%-CI: 2.33–4.24), the number of previous child victims (estimate = 0.52, 95%-CI: −0.18–1.21) and the MSI subscale “sexual knowledge and beliefs” (estimate = 0.16, 95%-CI: 0.06–0.25). Furthermore, this regression model explained 88.5% of the variance in the decision scores (adjusted *R*^2^= 0.885).

**TABLE 4:** Stepwise linear regression results.

	Estimate	95% CI	SE	*T*	*P*
Intercept	−6.87	[−9.01, −4.73]	0.95	−7.27	4.74E-05
SVR-20 final assessment score	3.28	[2.33, 4.24]	0.42	7.78	2.77E-05
Number of previous victims	0.52	[−0.18, 1.21]	0.31	1.69	0.13
MSI: sexual knowledge and beliefs	0.16	[0.06, 0.25]	0.04	3.65	0.01
SVR-20: Number of victims	−0.40	[−0.76, −0.05]	0.16	−2.58	0.03

SE, standard error; T, T statistic; P, *P*-value; SVR-20, Sexual Violence Risk-20; MSI, Multiphasic Sex Inventory; CI, confidence interval.

## Discussion

In this pilot study, we used DTI data and supervised machine learning to generate a brain-based predictive model for pedophilic offenders (PO). We combined the efficacy of linear SVM at low sample sizes with a literature-based region of interest approach, focusing on key brain areas in the prefronto-temporo-limbic circuit that have previously been implicated in pedophilia, sexual behavior, and pedophilic offending (10, 20, 21). The final PO model discriminated PO from HC individuals with 75.5% BAC and produced an out-of-sample specificity of 94.3% in HC individuals. In the final PO model, higher FA bilaterally in the pgACC as well as in the right dACC and rACC was highly predictive of PO individuals. In contrast, higher MD and RD in the left amygdala were among the strongest predictors of HC individuals. The AD domain did not contribute decisively to the overall prediction. SC measures showed hemispherical polarity, since higher SC in the left hemisphere (amygdala to PFC and vice versa) was predictive of PO individuals, while higher SC in the right hemisphere (amygdala to PFC) contributed to the prediction of HC. Overall, bilateral FA in the pgACC as well as bilateral amygdala to PFC connectivity were the four most consistent predictors and the most reliable discriminators between HC and PO.

The DTI parameters used in this study are sensitive to certain aspects of white matter (WM) microarchitecture (63). Specifically, increased myelination, fiber density, and axon coherence are associated with higher anisotropy (FA) and lower diffusivity measures (MD, RD, AD) (64).

The development of WM microstructure follows a distinct trajectory throughout the life of an individual and is strongly linked to cognitive abilities, social behavior, language skills, and motor function (64–66). Initially, rapid increases in brain organization occur during childhood and adolescence, followed by a slowing of the development process in young adulthood, before reaching a maturational peak, reversing trend, and declining in later adulthood and old age (65). Thus, FA increases steadily until the late 20 s and then decreases after the age of 30. Diffusivity measures follow a reverse profile, with decreases in MD and RD until the mid to late 30 s and a subsequent increase thereafter. AD follows a slightly prolonged pattern, reaching its minimum in the 40 s (65). This leads to a non-linear “U-shaped” trajectory for diffusivity and an “inverted U-shaped” trajectory for anisotropy measures (65). The main WM tracts show similar maturation patterns (65). Specifically, the uncinate fasciculus (UF), which connects the PFC and amygdala, reaches its maturational peak in the mid to late 30 s and declines thereafter (67).

This development of WM microstructure is a multifactorial process (64). The general trajectory of WM microstructure development is shaped and to some extent predetermined by genetics (68, 69). However, the genetic influence on WM microstructure decreases with age as environmental factors become increasingly important (64, 68, 70). Among these factors, positive influences such as breastfeeding and nutritional support are related to faster and/or greater WM development, while negative influences that include prenatal exposures (e.g., parental alcohol consumption, anxiety, or depression) or adverse childhood experiences (i.e., deprivation, neglect) appear to cause slower or impaired WM development (64). Furthermore, an earlier development of WM in girls and a more prolonged one in boys during puberty suggest that the genotype of androgen receptors also influences the longitudinal trajectory of WM development (71, 72). Therefore, the predictive WM microstructure pattern is likely also influenced by these factors and highlights the complexity of such a structural brain signature. Additionally, it emphasizes that a complex interplay of genetic, hormonal, and environmental factors is likely shaping this WM signature of high-risk PO individuals.

This is supported by a large body of evidence reporting particularly higher loads of hormonal and environmental risk factors in pedophilic men, but even more so in PO individuals (10, 73–75). In particular, higher rates or prenatal and perinatal influences, such as prenatal androgenization or parent psychopathology, have been identified in PO individuals (76–79). Furthermore, a strong association has been established between childhood trauma and stronger pedophilic interest, higher rates of sexual offending, and targeting of younger victims (80–84). Therefore, it could be speculated that the WM signature of PO individuals is the neurobiological correlate of multiple components exceeding a threshold above which pedophilic offending behavior becomes increasingly possible (76).

Detecting this signature, particularly in the microstructure of WM, is of great forensic relevance due to the strong involvement of WM maturation and development in the context of higher-order cognitive abilities and behaviors, which could all play a role in the enactment of pedophilic impulses and the commission of pedosexual crimes (64–66). WM microstructure in the frontoparietal circuit is closely related to reasoning, that is, the capacity to solve problems in novel situations (85). FA in the frontal and limbic lobe has been associated with inhibitory behavior, i.e., the ability to withhold an automatic response, resist tempting behavior, or adjust behavior to meet situational demands (86). Another important complex cognitive function is delay gratification, which defines the ability to weigh a preference for a larger delayed reward compared to a smaller, more immediate reward. This ability, which is highly dependent on impulse regulation and reward processing, has been linked to FA, diffusivity, and connectivity patterns in the frontostriatal tract and several other fasciculi, including the uncinate fasciculus (UF) (87–89). Finally, another complex cognitive behavior, which may be relevant in the forensic context, is risky decision making. Studies have repeatedly shown that, especially in adolescents and young adults, the microstructure of WM in the corpus callosum was related to levels of risk taking (90–93). Thus, WM microstructure integrity and deviations in WM development have been linked to various complex cognitive abilities, most of which relate to higher-order cognitive control, reward processing, and impulse regulation, all of which are highly relevant in the forensic setting (14, 40, 94–96).

However, the regions of interest chosen in this study are also highly involved in the generation and control of sexual arousal (20). While the ACC and the amygdala are responsible for the attribution of salience and impulse generation with respect to sexual stimuli, the PFC suppresses the output of the amygdala in a top-down manner through the UF (38). WM disruptions in the UF have been associated with deficits in empathic capacity and social cognition (39). Low empathy has long been discussed as a dynamic risk factor for child sexual offending, potentially separating offending from non-offending pedophilic individuals (40, 41). However, in a more recent meta-analysis, low victim empathy had little to no relationship with recidivism in persistent sexual offenders (40). Our findings, linking the UF to PO individuals, suggest that a more in-depth neurobiological investigation of the relationship between empathy and child sexual offending could lead to a better understanding of how these two phenomena are intertwined.

In summary, our findings, specifically the increase in FA in the pgACC and the increased structural amygdala to PFC connectivity, support a pathophysiological model of disinhibited aberrant sexual impulses and deficient higher-order cognitive control in PO individuals. In this model, dysfunctional ACC and amygdala signaling could lead to pedophilic arousal and the generation of pedosexual behavioral impulses, which are not sufficiently controlled, as the amygdala overrides prefrontal control in a bottom-up manner. However, our current knowledge on WM suggests that this aberrant pattern of WM microstructure could potentially be influenced by learning and/or intense activity even in adulthood (97). Therefore, dysfunctional behavior, such as pre-occupation with sexual pedophilic themes, continuous consumption of child pornography, or even pedophilic reoffending, could possibly further negatively impact or strengthen this WM microstructure pattern. In contrast, lifestyle changes and psychotherapeutic interventions such as the BEDIT program (98), the Good Lives Model (95), or other types of psychosocial support could potentially lead to behavioral changes, which might eventually be reflected in changes in the WM microstructure pattern.

The *post hoc* investigation pointed to the possible clinical and forensic utility of such a brain-based PO biomarker. Using stepwise linear regression, the PO classifier scores were predicted by the number of previous child victims, the MSI subscale “sexual knowledge and beliefs,” as well as the final assessment score in the SVR-20, with the latter reflecting the assessment by a mental health professional of the risk of future sexual violent reoffences. We therefore assume that the model indeed captured a WM microstructure pattern which is potentially modifiable and appears to be closely related to past child sexual offending, current stance on sexuality, and risk of future sexual violent reoffending.

Since this model was developed in an evenly distributed sample of PO and HC individuals, we assessed the added value of using such predictive tools in possible real-world scenarios, where the rate of PO individuals is highly dependent on the selection of individuals, i.e., the context in which such a tool is used (7). While the incidence of pedophilia is estimated to be 1% in the general population, the incidence of PO individuals is much more difficult to assess. In representative community studies, self-reported abuse of a child ranged from 0.05 to 4% (7, 12, 99). However, in more selective high-risk subpopulations containing individuals with self-reported sexual interest in children, the prevalence of self-reported actual sexual contact with children was reported to be between 39.4 (100) and 50% (98, 101). Moreover, the relapse rates for previously convicted pedophilic offenders range from 50 to 80% (102). Therefore, depending on the setting, the model could face highly varying degrees of prevalence rates of PO individuals. Thus, it is encouraging that the model performed robustly at prevalence rates of PO individuals between 20 and 70% with PSI values greater than 55% and an NNP less than 3. Therefore, the model would increase diagnostic certainty in cohorts with these prevalence rates by more than 55%, compared to the level of simple chance (52, 103). Furthermore, no more than three people in these cohorts would have to be tested with this model to correctly identify one PO individual. Although the model still produced a solid PSI of 18% and an NNP of 5.51 at a lower prevalence rate of 5%, it became clear that this model is most well suited to be applied to clinically preselected high-risk cohorts with expected higher rates of PO individuals. Examples of this could be individuals with self-reported interest in children, a diagnosis of pedophilia, or individuals who have already committed pedophilic offenses. Hence, a model like this could provide additional certainty when applied to individuals that have already been preselected by mental health professionals or other professionals in the field of forensic psychiatry.

The most important limitation of this study is the small sample size of 29 subjects in the main study sample and 53 subjects in the external HC sample (104). Machine learning algorithms provide high predictive potential because they can discover subtle distributed effects that would otherwise not be detected by conventional univariate analysis (105). However, this enhanced pattern recognition makes machine learning algorithms also susceptible to certain issues. First, they can provide misleading results when used on small samples since small samples carry the risk of not being representative of the population of interest and instead being more defined by specific batch effects (106, 107). These batch effects can stem from the catchment area, the recruitment period, the recruiting personnel, and many more factors that can heavily confound the information contained in a sample. When working with small study samples, these algorithms might discover patterns that at first glance seem to be related to the desired phenotype but are heavily confounded by these much more profane factors related to specific characteristics of the study samples. Second, machine learning algorithms tend to overfit in small samples. Depending on the setup of the learning parameters, i.e., hyperparameters, machine learning algorithms create a model which closely fits the characteristics of the study sample. However, when the sample is small and not representative of the phenotype of interest in the real-world populations, then the resulting model is “overfitted” to even minor details, trivial characteristics, or even random effects of the study sample (108). This can eventually lead to the phenomenon that a model performs well in the study sample, but then fails to detect a certain phenotype in new and previously unseen individuals. This lack of generalizability due to overfitting is one of the major concerns in machine learning and appears frequently in models trained on smaller samples. A third issue arises from possible discrepancies between the frequency of a desired phenotype in the study sample compared to the general population. Therefore, a model trained on a carefully selected sample in which a certain phenotype is highly enriched and therefore equally distributed compared to healthy control individuals could perform rather poorly when applied to more naturalistic general populations, in which the phenotype is much scarcer (109). This problem can be further impeded when the targeted phenotype exerts a massive, sometimes irreversible, impact on the affected individual or its surroundings. When a machine learning model, which was trained to predict response to antidepressant treatment, predicts a wrong outcome, it can lead to a delay in therapy response and, for example, to a potentially longer hospitalization or sick leave. However, when a machine learning model, which was trained to predict pedophilic offending, fails, and incorrectly classifies an individual as “low risk”, which then proceeds to commit such an offense, the damage is much greater. Our study investigates a phenotype, pedophilic offending, which is, on the one hand, very rare in the general population and, on the other hand, devastating if misclassified. Therefore, the results of our study must be cautiously interpreted as they were derived from a small single-center cohort and the final model predicts a phenotype that is both rare and highly impactful in the general population. Faced with these difficulties regarding interpretability and generalizability, we took some steps to at least mitigate these issues. First, we used a repeated nested cross-validation approach with 10-folds and 10 permutations each on the CV2 and CV1 level. Therefore, despite our small sample, a total of 10,000 models were computed, and the results presented here (CV ratio, sign-based consistency, balanced accuracy) are the aggregated across all these iterations. This approach has been shown to robustly prevent overfitting and increase generalizability, even when faced with small sample sizes (25, 110). Furthermore, we used a machine learning algorithm, the support vector machine with a linear kernel, which has been repeatedly shown to perform particularly well at small sample sizes (49). In addition, we added advanced performance metrics (PSI, NNP) to investigate how our PO model would perform when faced with varying rates of PO individuals in possible real-world scenarios. Finally, while a true replication sample containing both HC and PO individuals was not available, we used an external sample of HC individuals, in which our PO model showed high specificity for correctly detecting HC and not misdiagnosing them as PO individuals.

Beyond these more methodical elaborations, further limitations of our study are related to the intricacies of the study populations. Our HC populations from both samples were non-offending, non-pedophilic individuals, whereas non-pedophilic child sexual offenders or non-offending pedophilic individuals would have been more specific control groups. Our PO population was an inpatient cohort of hands-on pedophilic offenders without any comorbid psychiatric disorders, which carries additional limitations. First, the inpatient status could mean that certain effects of deprivation or incarceration might have influenced the results. Second, our study sample did not include hands-off pedophilic offenders, namely consumers of media depicting child sexual abuse, which is an important mode of child sexual offending leading to the victimization of a great number of children (111, 112). Third, excluding PO individuals with comorbid psychiatric diagnoses might have decreased the representative quality of the study sample since PO individuals in the general population often suffer from multiple mental health issues (113). Due to these limitations, the PO model generated in this study should be considered strictly exploratory in nature and is not meant to be translated immediately into clinical practice. Rather, our approach of using machine learning and neurobiological features is supposed to serve as a template for future endeavors regarding biomarker-enhanced risk assessment in the field of CSA and forensic psychiatry. Future studies should build on our pilot study using higher resolution magnetic resonance imaging, larger samples, tailored control groups, and a more in-depth sociodemographic, clinical, and forensic assessment.

To our knowledge, this is the first study to successfully train a supervised machine learning classifier on WM microstructure patterns to distinguish between PO and HC individuals. The brain-based PO model produced a BAC of 75.5%, an out-of-sample specificity of 94.3% and was related to the individual’s previous number of child victims, current stance on sexuality, and future risk of sexual violent reoffending. We hypothesize that the discriminatory pattern of WM in the amygdala, ACC, and PFC could reflect a high-risk interaction between genetic predisposition, hormonal influence, and environmental factors throughout an individual’s life. We propose this WM microstructure pattern in the prefronto-temporo-limbic circuitry as a template for further research on brain-based biomarkers for pedophilic offenders and we hope that such biomarkers will improve secondary and tertiary prevention of child sexual abuse.

## Data availability statement

The raw data supporting the conclusions of this article will be made available by the authors, without undue reservation.

## Ethics statement

The studies involving human participants were reviewed and approved by the local ethics advisory board of the Medical School at Otto-von-Guericke University, Magdeburg. The patients/participants provided their written informed consent to participate in this study.

## Author contributions

DP and KS had full access to all the data in the study, took responsibility for the integrity of the data and the accuracy of the data analysis, and contributed to the concept and design of the study. DP, MWe, CG, JK, KS, MWa, and JW contributed to the acquisition, analysis, or interpretation of data. DP, MWe, CG, and JK drafted the manuscript. DP, MWe, ML, JL, NK, KS, PF, BB, and MWe contributed to the critical revision of the manuscript for important intellectual content. DP, MWe, JK, and NK contributed to the statistical analysis. DP, KS, JW, and BB obtained the funding. PF, JK, KS, BB, JW, and NK contributed to the administrative, technical, or material support. PF, NK, and KS contributed to the supervision. All authors contributed to the article and approved the submitted version.

## Footnotes

1
http://surfer.nmr.mgh.harvard.edu/

2
http://proniapredictors.eu/neurominer/index.html
