# Supplementary material for: Patterns of risk—Using machine learning and structural neuroimaging to identify pedophilic offenders
Source: Front Psychiatry. 2023 Apr 20;14:1001085. doi: 10.3389/fpsyt.2023.1001085 (PMC10157073; doi:10.3389/fpsyt.2023.1001085)
Supplement: Supplementary file 1 [file Data_Sheet_1.DOCX]

Patterns of risk - Using Machine Learning and Structural Neuroimaging to Identify Pedophilic Offenders

David Popovic^1,2^, Maximilian Wertz^1^, Carolin Geisler^3^, Joern Kaufmann^4^, Markku Lähteenvuo^5,6^, Johannes Lieslehto^5^, Joachim Witzel^7^, Bernhard Bogerts^8,9^, Martin Walter^10^, Peter Falkai^1,2^, Nikolaos Koutsouleris^1,2,11^, Kolja Schiltz^1^

1. Department of Psychiatry and Psychotherapy, Ludwig-Maximilian-University, Munich, Germany
2. International Max Planck Research School for Translational Psychiatry (IMPRS-TP), Munich, Germany

1. [Department of Dermatology, Venereology and Allergology](https://derma.charite.de/en/), Charité – Universitätsmedizin Berlin, Germany
2. Department of Neurology, Otto-von-Guericke-University, Magdeburg, Germany
3. Department of Forensic Psychiatry, University of Eastern Finland, Niuvanniemi Hospital, Kuopio, Finland
4. Institute for Molecular Medicine FIMM, University of Helsinki, Helsinki, Finland
5. Central State Forensic Psychiatric Hospital of Saxony-Anhalt, Uchtspringe, Germany
6. Salus Institut, Salus gGmbH, Magdeburg, Germany
7. Department of Psychiatry and Psychotherapy, Otto-von-Guericke-University, Magdeburg, Germany
8. Department of Psychiatry and Psychotherapy, Jena University Hospital, Jena, Germany
9. Institute of Psychiatry, Psychology and Neuroscience, King’s College London, London, United Kingdom

**Keywords:** pedophilia, MRI, child sexual abuse, support vector machines, forensic psychiatry, machine learning

*Supplementary Material*

# Supplementary Methods

## MRI data acquisition

MRI data from the main study and the external HC sample were acquired using a General Electric Signa LX 1.5-T system (General Electric, Milwaukee, WI, USA) with actively shielded magnetic field gradients (maximum amplitude 40 mT m^-1^). A standard quadrature birdcage head coil was used for both radio frequency transmission and signal reception. The MR protocol included a T1-weighted sagittal 3D scan (contrast-optimized spoiled gradient-echo sequence; 124 slices, slice thickness 1.5 mm; TE 8 ms; TR 24 ms; flip angle 30 degree) and a single-shot diffusion-weighted spin-echo-refocused echo planar imaging sequence (data acquisition matrix: 128 x 128; field of view 280 mm x 280 mm; TE 70 ms; TR 10.000 ms; 39 slices; slice thickness 3 mm; b-value 1,000 s/mm^2^).

Fiber tract reconstruction was carried out using a previously described double-step probabilistic approach (1). In summary, a Monte Carlo simulation algorithm was implemented in Matlab that repeatedly searches for probable paths through the derived diffusion tensor matrix. Starting from voxels in a predefined start region, each single path propagation step is calculated on the basis of the diffusion tensors of the candidate successor voxels. An estimate of the voxel-specific probability distribution of axonal connections is used to calculate the probabilities of all allowable propagation steps. Explicitly, drawing randomly from this distribution determines each step. A full path was terminated if the apparent diffusion coefficient exceeded a threshold of 1.8 x 10^-9^ m^2^/s. This ensured that the putative tracks were located within the neural tissue.

As seed and target regions, automatically segmented amygdala and prefrontal cortex (PFC) regions based on T1-weighted volumes and generated by the freesurfer recon-all pipeline (freesurfer version 6.0.0 (2)) were used (<http://surfer.nmr.mgh.harvard.edu/>). Subcortical labels 18 and 54 for the left and right amygdala, respectively, and a list of cortical labels were used to build binary masks with the FSL tool fslmaths (3). The regions were coregistered to the diffusion data using the FSL tools flirt and fnirt, and the fiber tracking was performed in the diffusion space. Tractographic analyses between seed and target regions were carried out in both hemispheres with 5000 starts per voxel within the start region. Tractography was performed bidirectionally and all detected paths were stored. Fiber counts were normalized by division with the total number of starts from each start region and are measures of structural connectivity.

## Machine Learning Pipeline

In order to ensure model generalizability and prevent information leakage, we used a nested cross-validation (NCV) framework (4,5), which robustly prevents information leakage between individuals used to train and test the models (6). We split the data first into training and test folds on the outer (CV2) cycle, and then we split the resulting training folds again into 10 inner (CV1) training and testing folds, which leads to a 10-by-10-fold NCV (4,7,8). Hyperparameter optimization is performed on the CV1 cycle, whereas model testing is performed only on the CV2 cycle. CV2 samples never came into contact with the classification algorithms during the training process (6). Hence, all model training steps that use group-level statistical procedures (e.g. scaling, pruning, wrapper-based feature selection) occur only in the CV1 cycle training data. The CV1 cycle test data are used to pick hyperparameter combinations that provide good model generalization capacity. Finally, CV2 test data are used to measure the generalizability of the models to new unseen data. We extended NCV to repeated NCV (9) at both CV2 and CV1 levels by randomly permuting participants within their groups (number of permutations = 10) and repeating the CV cycle for each of these permutations. Our NeuroMiner machine learning pipeline (v1.05;<https://github.com/neurominer-git>) consisted of the following steps:

1. Each variable was scaled to a 0-1 range, while non-informative features (zero variance) were removed.
2. Since HC and PO subjects did not differ significantly for age and education years, no further correction of the dataset was employed (10,11).
3. The classifier then underwent a stepwise forward variable selection process (11) using a linear support vector machine (SVM) (12). The procedure grew into a parsimonious combination of features that optimized the average classification performance of the algorithm across the CV1 training and testing data. In doing so, data from every modality entered a greedy forward search wrapper (11), which allows identification of the most parsimonious subset of variables within the variable pool of every modality. This provides maximum prognostic performance with the smallest amount of predictive features. Within each classifier, the wrapper algorithm used an SVM to evaluate the predictive value of each variable within the modality, then extracted the most predictive variable, and reiterated over the remaining variable pool to select the second best performing variable, which was added to the first one. This process was repeated until the optimal variable subspace was identified. We stopped the variable search when 90% of the top variables had been extracted by the wrapper, allowing us to identify a clinically applicable set of top-performing variables within every classifier for prediction purposes. The wrappers added single features up to 10% of the total and then tested the models.

The trained model was applied to the corresponding CV2 fold by preprocessing the best discriminative variables using the learned scaling of the CV1 cycle and determining the outcome class of each individual tested (HC vs. PO) by majority voting in all ensemble models. In other words, in each variable evaluation step in CV1, the SVM algorithm modeled linear relationships between features and classification labels (HC vs PO). In the linear kernel space, the SVM optimized a hyperplane that maximized separability between most HC-like and most PO-like subjects (i.e., the support vectors). Based on the trained hyperplane, the algorithm then predicted the subjects' classification of the inner CV1 cycle by projecting its data into the learned kernel space and measuring their geometric distance to the decision boundary. This resulted in a decision value and a predicted classification label per subject. In uneven group sizes at the CV1 level, hyperplane weighting was activated. The wrapper-based feature selection was carried out for each CV1 training and test sample and then repeated for every combination of the SVM parameters C (misclassification cost) and γ (kernel width) within a grid defined by the ranges C = [0.0156 - 16] and γ = [3.0518-5 - 8]. Because of our repeated nested cross-validation framework, we created an ensemble of 100 models (n=10 repetition x k=10 folds) for each CV2 partition (‘CV1 ensemble’). Furthermore, due to the 10 repetitions of the CV2 cycle, we were able to establish a final out-of-training class membership prediction for a given individual by combining all CV1 ensembles into a larger CV2 ensemble, in which the given individual had not served for model training and optimization at the CV1 level. This ensemble generation procedure has been repeatedly described in our previous work (13) and is a feature of the model generation and validation process implemented in NeuroMiner.

## Permutation testing

To assess the statistical significance of the final PO model, we employed permutation testing (14). We performed 5000 random permutations of the outcome labels. For each permutation, we retrained the PO model in the repeated NCV structure using the respective feature subsets obtained from the observed-label analyses. For each permutation, we accumulated the predictions of the random models into a permuted ensemble prediction for each CV2 subject. Thus, we built a null distribution of out-of-training classification performance (BAC). We then calculated the significance of the observed out-of-training BAC as the number of events in which the permuted out-of-training BAC was greater than or equal to the observed BAC divided by the number of permutations performed. The significance of the model was determined at q=0.05.

## Software

All analyses were conducted using Matlab R2020b (MathWorks, Natick, MA, USA). Specifically, we used functions from the Matlab Statistics and Machine Learning Toolbox as well as the machine learning software NeuroMiner, version 1.05 developed by Nikolaos Koutsouleris (<http://proniapredictors.eu/neurominer/index.html>) (4,15). 3D brain renderings were generated via the open-source software MRIcroGL (McCausland Center for Brain Imaging, University of South Carolina; <https://www.nitrc.org/projects/mricrogl/>). DTI data analysis was performed on the Freesurfer platform (freesurfer version 6.0.0 (2), <http://surfer.nmr.mgh.harvard.edu/>) and the FSL software library ((3) <https://fsl.fmrib.ox.ac.uk/fsl/fslwiki/>).

# Supplementary Tables

**Table S1:Multiphasic Sex Inventory scores of the forensic sample**

| **Parent subtest** | **Superordinate scale** | **Subscale** | **Mean** | **SD** |
| --- | --- | --- | --- | --- |
| Validity  Scales |  | Social sexual desirability | 25.86 | 7.42 |
|  |  | Sexual compulsivity | 5.36 | 4.88 |
|  | Lie scales | Sexual abuse of children | 3.93 | 2.55 |
|  |  | Rape | 10.71 | 2.31 |
|  |  | Exhibitionism | 4.57 | 0.82 |
|  |  | Incest | 2.50 | 0.73 |
|  |  | Cognitive bias and immaturity | 9.36 | 4.42 |
|  |  | Justification | 7.07 | 4.48 |
|  |  | Opinion on the treatment | 4.86 | 1.36 |
| Course and behavior patterns of  sexual deviance | Child sexual abuse | **Total score** ^a^ | 21.93 | 5.69 |
|  |  | Fantasy ^a^ | 6.07 | 2.28 |
|  |  | Searching/sneaking around and persuasion tactics ^a^ | 5.43 | 2.26 |
|  |  | Sexual assault/attack ^a^ | 6.71 | 1.48 |
|  |  | Aggravated sexual assault | 2.93 | 1.49 |
|  |  | Incest | 0.79 | 1.01 |
|  |  | Abuse of girls | 2.00 | 1.20 |
|  |  | Abuse of boys | 1.50 | 1.05 |
|  | Rape | **Total score** | 3.79 | 6.17 |
|  |  | Fantasy | 0.93 | 1.28 |
|  |  | Searching/sneaking around | 0.86 | 1.46 |
|  |  | Sexual assault/attack | 0.93 | 1.44 |
|  |  | Aggravated sexual assault | 0.57 | 0.73 |
|  |  | Sadomasochism | 0.50 | 1.80 |
|  | Exhibitionism | **Total score** | 1.79 | 1.26 |
|  |  | fantasy | 0.29 | 0.59 |
|  |  | Searching/sneaking around | 0.43 | 0.49 |
|  |  | Sexual assault/attack | 1.00 | 0.26 |
|  |  | Advanced Exhibitionism | 0.07 | 0.26 |
| Paraphilias | | **Total score** ^a^ | 3.50 | 3.64 |
|  |  | Fetishism | 0.79 | 0.94 |
|  |  | Voyeurism ^a^ | 1.57 | 1.64 |
|  |  | Obscene phone calls | 0.14 | 0.35 |
|  |  | Bondage and discipline | 0.36 | 0.72 |
|  |  | Sadomasochism | NA | NA |
| Sexual dysfunctions | | **Total score** | 4.93 | 2.99 |
|  |  | Sexual inadequacy | 1.86 | 1.25 |
|  |  | Premature ejaculation | 0.50 | 0.73 |
|  |  | Physical disability | 0.43 | 0.73 |
|  |  | Impotence | 2.14 | 2.03 |
| Sexual knowledge & beliefs | | **Total score** | 15.93 | 3.67 |

^a^ above average results in comparison to a German norm sample of child abuser (n = 230)(16)

**Table S2: MNI coordinates (center) of the spherical anterior cingulate cortex (ACC) sub-regions** (18,19) **and spherical amygdala regions** (20)**.**

Abbreviations: cACC, caudal anterior cingulate cortex; dACC, dorsal anterior cingulate cortex; rACC, rostral anterior cingulate cortex; pgACC, pregenual anterior cingulate cortex; sgACC, subgenual anterior cingulate cortex.

|  | Left (x, y, z) in mm | Right (x, y, z) in mm |
| --- | --- | --- |
| cACC | -5, -10, 47 | 5, -10, 47 |
| dACC | -5, 14, 42 | 5, 14, 42 |
| rACC | -5, 34, 28 | 5, 34, 28 |
| pgACC | -5, 47, 11 | 5, 47, 11 |
| sgACC | -5, 25, -10 | 5, 25, -10 |
| amygdala | -20, -4, -16 | 24, -4, -16 |

**Table S3: Group-level differences of WM microstructure features in the main study sample.**

U: Test statistic, Mann-Whitney-Test. Results are stated as mean value followed by its standard deviation in brackets: μ (SD). All P values FDR-adjusted for multiple testing using all P values from the table as a family of tests (17). Abbreviations: AD, Axial Diffusivity; FA, fractional anisotropy; MD, Mean Diffusivity; RD, Radial Diffusivity; SC, structural connectivity; HC, healthy control subjects; cACC, caudal anterior cingulate cortex; dACC, dorsal anterior cingulate cortex; rACC, rostral anterior cingulate cortex; pgACC, pregenual anterior cingulate cortex; sgACC, subgenual anterior cingulate cortex; CC, corpus callosum; PFC, prefrontal cortex.

|  | Left hemisphere | | | | | Right hemisphere | | | | |
| --- | --- | --- | --- | --- | --- | --- | --- | --- | --- | --- |
|  | **AD in 10^-9^ m^2^/s** | | | | | | | | | |
|  | All | HC | PO | U | P | All | HC | PO | U | P |
| cACC | 1.05 (0.17) | 1.02 (0.1) | 1.08 (0.23) | -0.94 | 0.70 | 1.05 (0.19) | 1.03 (0.07) | 1.07 (0.27) | 0.37 | 0.88 |
| dACC | 1.08 (0.16) | 1.04 (0.09) | 1.11 (0.22) | -0.63 | 0.86 | 1.12 (0.26) | 1.14 (0.22) | 1.1 (0.31) | 0.94 | 0.70 |
| rACC | 1.11 (0.23) | 1.09 (0.23) | 1.13 (0.23) | -1.37 | 0.70 | 1.09 (0.19) | 1.1 (0.18) | 1.08 (0.2) | 0.41 | 0.87 |
| pgACC | 1.12 (0.14) | 1.11 (0.15) | 1.13 (0.13) | -0.76 | 0.80 | 1.17 (0.23) | 1.17 (0.21) | 1.17 (0.26) | 0.46 | 0.87 |
| sgACC | 1.03 (0.05) | 1.02 (0.04) | 1.04 (0.06) | -1.03 | 0.70 | 1.02 (0.07) | 1.02 (0.04) | 1.01 (0.1) | 1.94 | 0.61 |
| amygdala | 0.94 (0.03) | 0.95 (0.03) | 0.94 (0.03) | 1.03 | 0.70 | 0.97 (0.06) | 0.97 (0.05) | 0.97 (0.08) | 0.98 | 0.70 |
|  | All | | HC | | PO | | U | | P | |
| CC segment 1 | 1.65 (0.07) | | 1.66 (0.07) | | 1.65 (0.08) | | 0.41 | | 0.87 | |
| CC segment 2 | 1.87 (0.26) | | 1.86 (0.25) | | 1.88 (0.27) | | -0.11 | | 0.97 | |
| CC segment 3 | 2.29 (0.34) | | 2.34 (0.27) | | 2.23 (0.4) | | 0.98 | | 0.70 | |
| CC segment 4 | 2.31 (0.36) | | 2.36 (0.26) | | 2.26 (0.44) | | 1.16 | | 0.70 | |
| CC segment 5 | 1.74 (0.1) | | 1.74 (0.11) | | 1.74 (0.08) | | -0.15 | | 0.97 | |
|  |  | |  | |  | |  | |  | |
|  | **FA** | | | | | | | | | |
|  | All | HC | PO | U | P | All | HC | PO | U | P |
| cACC | 0.13 (0.03) | 0.12 (0.02) | 0.14 (0.03) | -1.33 | 0.70 | 0.14 (0.04) | 0.13 (0.03) | 0.14 (0.05) | -1.24 | 0.70 |
| dACC | 0.13 (0.02) | 0.13 (0.01) | 0.13 (0.02) | -0.02 | 0.98 | 0.13 (0.02) | 0.12 (0.02) | 0.14 (0.03) | -2.07 | 0.61 |
| rACC | 0.13 (0.02) | 0.13 (0.02) | 0.14 (0.03) | -1.77 | 0.61 | 0.13 (0.03) | 0.12 (0.02) | 0.14 (0.03) | -1.94 | 0.61 |
| pgACC | 0.14 (0.03) | 0.13 (0.02) | 0.16 (0.03) | -2.55 | 0.61 | 0.13 (0.02) | 0.12 (0.02) | 0.14 (0.02) | -2.38 | 0.61 |
| sgACC | 0.2 (0.03) | 0.2 (0.03) | 0.21 (0.04) | -0.37 | 0.88 | 0.2 (0.03) | 0.2 (0.02) | 0.2 (0.03) | 0.50 | 0.87 |
| amygdala | 0.15 (0.02) | 0.15 (0.01) | 0.16 (0.02) | -1.85 | 0.61 | 0.16 (0.02) | 0.17 (0.02) | 0.16 (0.02) | 0.55 | 0.87 |
|  | All | | HC | | PO | | U | | P | |
| CC segment 1 | 0.6 (0.06) | | 0.61 (0.05) | | 0.59 (0.07) | | 0.85 | | 0.73 | |
| CC segment 2 | 0.38 (0.09) | | 0.38 (0.09) | | 0.38 (0.08) | | -0.11 | | 0.97 | |
| CC segment 3 | 0.35 (0.08) | | 0.33 (0.06) | | 0.37 (0.1) | | -1.11 | | 0.70 | |
| CC segment 4 | 0.34 (0.1) | | 0.32 (0.07) | | 0.37 (0.12) | | -0.89 | | 0.70 | |
| CC segment 5 | 0.5 (0.08) | | 0.49 (0.07) | | 0.51 (0.08) | | -0.89 | | 0.70 | |
|  |  | |  | |  | |  | |  | |
|  | **MD in 10^-9^ m^2^/s** | | | | | | | | | |
|  | All | HC | PO | U | P | All | HC | PO | U | P |
| cACC | 0.92 (0.14) | 0.9 (0.11) | 0.93 (0.17) | -0.50 | 0.87 | 0.91 (0.14) | 0.9 (0.09) | 0.93 (0.18) | -0.11 | 0.97 |
| dACC | 0.95 (0.15) | 0.92 (0.1) | 0.99 (0.19) | -0.72 | 0.81 | 0.98 (0.23) | 1.02 (0.21) | 0.94 (0.26) | 1.07 | 0.70 |
| rACC | 0.97 (0.2) | 0.96 (0.23) | 0.98 (0.16) | -1.29 | 0.70 | 0.96 (0.16) | 0.98 (0.18) | 0.94 (0.15) | 0.59 | 0.87 |
| pgACC | 0.97 (0.13) | 0.97 (0.14) | 0.97 (0.11) | -0.28 | 0.95 | 1.04 (0.21) | 1.04 (0.19) | 1.03 (0.23) | 0.41 | 0.87 |
| sgACC | 0.82 (0.03) | 0.81 (0.02) | 0.83 (0.04) | -1.42 | 0.70 | 0.82 (0.04) | 0.82 (0.03) | 0.82 (0.06) | 0.63 | 0.86 |
| amygdala | 0.8 (0.02) | 0.81 (0.03) | 0.79 (0.02) | 1.81 | 0.61 | 0.8 (0.04) | 0.8 (0.02) | 0.8 (0.05) | 1.35 | 0.70 |
|  | All | | HC | | PO | | U | | P | |
| CC segment 1 | 0.86 (0.06) | | 0.86 (0.05) | | 0.87 (0.07) | | -0.02 | | 0.98 | |
| CC segment 2 | 1.32 (0.31) | | 1.31 (0.31) | | 1.33 (0.32) | | -0.24 | | 0.96 | |
| CC segment 3 | 1.67 (0.38) | | 1.72 (0.3) | | 1.61 (0.45) | | 0.94 | | 0.70 | |
| CC segment 4 | 1.66 (0.4) | | 1.71 (0.29) | | 1.61 (0.5) | | 1.11 | | 0.70 | |
| CC segment 5 | 1 (0.12) | | 1 (0.13) | | 0.99 (0.1) | | 0.15 | | 0.97 | |
|  |  | |  | |  | |  | |  | |
|  | **RD in 10^-9^ m^2^/s** | | | | | | | | | |
|  | All | HC | PO | U | P | All | HC | PO | U | P |
| cACC | 0.86 (0.13) | 0.85 (0.12) | 0.87 (0.14) | -0.24 | 0.96 | 0.85 (0.12) | 0.84 (0.1) | 0.86 (0.14) | -0.07 | 0.97 |
| dACC | 0.89 (0.14) | 0.86 (0.1) | 0.93 (0.17) | -0.72 | 0.81 | 0.92 (0.22) | 0.96 (0.21) | 0.88 (0.23) | 1.20 | 0.70 |
| rACC | 0.9 (0.18) | 0.9 (0.22) | 0.9 (0.13) | -1.24 | 0.70 | 0.9 (0.15) | 0.93 (0.17) | 0.87 (0.13) | 0.89 | 0.70 |
| pgACC | 0.9 (0.13) | 0.91 (0.14) | 0.9 (0.11) | -0.11 | 0.97 | 0.97 (0.19) | 0.97 (0.18) | 0.97 (0.21) | 0.41 | 0.87 |
| sgACC | 0.73 (0.04) | 0.72 (0.02) | 0.74 (0.04) | -1.20 | 0.70 | 0.73 (0.04) | 0.73 (0.03) | 0.73 (0.04) | -0.55 | 0.87 |
| amygdala | 0.74 (0.02) | 0.75 (0.02) | 0.73 (0.02) | 2.03 | 0.61 | 0.73 (0.03) | 0.73 (0.02) | 0.74 (0.04) | 0.89 | 0.70 |
|  | All | | HC | | PO | | U | | P | |
| CC segment 1 | 0.53 (0.08) | | 0.52 (0.07) | | 0.54 (0.09) | | -0.59 | | 0.87 | |
| CC segment 2 | 1.06 (0.33) | | 1.05 (0.33) | | 1.07 (0.35) | | -0.07 | | 0.97 | |
| CC segment 3 | 1.37 (0.39) | | 1.43 (0.32) | | 1.31 (0.46) | | 0.98 | | 0.70 | |
| CC segment 4 | 1.36 (0.42) | | 1.41 (0.3) | | 1.29 (0.52) | | 1.03 | | 0.70 | |
| CC segment 5 | 0.7 (0.13) | | 0.71 (0.14) | | 0.69 (0.13) | | 0.50 | | 0.87 | |
|  |  | |  | |  | |  | |  | |
|  | **SC** | | | | | | | | | |
|  | All | | HC | | PO | | U | | P | |
| amygdala left to  PFC left | 965214.21  (414623.74) | | 812072.8  (325409.12) | | 1129294.29  (447418.94) | | -1.72 | | 0.61 | |
| PFC left to  amygdala left | 1838308.24  (936869.2) | | 1532179.87  (650077.34) | | 2166302.93  (1100629.73) | | -1.51 | | 0.70 | |
| amygdala right to  PFC right | 1183673.1  (481384.82) | | 1323929.8  (518967.85) | | 1033398.07  (402554.88) | | 1.51 | | 0.70 | |
| PFC right  to amygdala right | 2184214.17  (743364.8) | | 2188780.67  (814467.37) | | 2179321.5  (689755.09) | | 0.15 | | 0.97 | |

# References

1. Bodammer NC, Kaufmann J, Kanowski M, Tempelmann C. Monte Carlo-based diffusion tensor tractography with a geometrically corrected voxel-centre connecting method. Phys Med Biol [Internet]. 2009 Feb 21;54(4):1009–33. Available from: http://dx.doi.org/10.1088/0031-9155/54/4/013

2. Fischl B. FreeSurfer [Internet]. Vol. 62, NeuroImage. 2012. p. 774–81. Available from: http://dx.doi.org/10.1016/j.neuroimage.2012.01.021

3. Jenkinson M, Beckmann CF, Behrens TEJ, Woolrich MW, Smith SM. FSL [Internet]. Vol. 62, NeuroImage. 2012. p. 782–90. Available from: http://dx.doi.org/10.1016/j.neuroimage.2011.09.015

4. Koutsouleris N, Kahn RS, Chekroud AM, Leucht S, Falkai P, Wobrock T, et al. Multisite prediction of 4-week and 52-week treatment outcomes in patients with first-episode psychosis: a machine learning approach. Lancet Psychiatry [Internet]. 2016 Oct;3(10):935–46. Available from: http://dx.doi.org/10.1016/S2215-0366(16)30171-7

5. Koutsouleris N, Kambeitz-Ilankovic L, Ruhrmann S, Rosen M, Ruef A, Dwyer DB, et al. Prediction Models of Functional Outcomes for Individuals in the Clinical High-Risk State for Psychosis or With Recent-Onset Depression: A Multimodal, Multisite Machine Learning Analysis. JAMA Psychiatry [Internet]. 2018 Nov 1;75(11):1156–72. Available from: http://dx.doi.org/10.1001/jamapsychiatry.2018.2165

6. Ruschhaupt M, Huber W, Poustka A, Mansmann U. A compendium to ensure computational reproducibility in high-dimensional classification tasks. Stat Appl Genet Mol Biol [Internet]. 2004 Dec 19;3:Article37. Available from: http://dx.doi.org/10.2202/1544-6115.1078

7. Varma S, Simon R. Bias in error estimation when using cross-validation for model selection. BMC Bioinformatics [Internet]. 2006 Feb 23;7:91. Available from: http://dx.doi.org/10.1186/1471-2105-7-91

8. Varmuza K, Filzmoser P, Hilchenbach M, Krüger H, Silén J. KNN classification — evaluated by repeated double cross validation: Recognition of minerals relevant for comet dust [Internet]. Vol. 138, Chemometrics and Intelligent Laboratory Systems. 2014. p. 64–71. Available from: http://dx.doi.org/10.1016/j.chemolab.2014.07.011

9. Dwyer DB, Falkai P, Koutsouleris N. Machine Learning Approaches for Clinical Psychology and Psychiatry. Annu Rev Clin Psychol [Internet]. 2018 May 7;14:91–118. Available from: http://dx.doi.org/10.1146/annurev-clinpsy-032816-045037

10. Weiss E, Siedentopf CM, Hofer A, Deisenhammer EA, Hoptman MJ, Kremser C, et al. Sex differences in brain activation pattern during a visuospatial cognitive task: a functional magnetic resonance imaging study in healthy volunteers. Neurosci Lett [Internet]. 2003 Jul 3;344(3):169–72. Available from: http://dx.doi.org/10.1016/s0304-3940(03)00406-3

11. Saeys Y, Inza I, Larrañaga P. A review of feature selection techniques in bioinformatics. Bioinformatics [Internet]. 2007 Oct 1;23(19):2507–17. Available from: http://dx.doi.org/10.1093/bioinformatics/btm344

12. Noble WS. What is a support vector machine? Nat Biotechnol [Internet]. 2006;24(12):1565–7. Available from: https://www.nature.com/articles/nbt1206-1565

13. Koutsouleris N, Riecher-Rössler A, Meisenzahl EM, Smieskova R, Studerus E, Kambeitz-Ilankovic L, et al. Detecting the psychosis prodrome across high-risk populations using neuroanatomical biomarkers. Schizophr Bull [Internet]. 2015 Mar;41(2):471–82. Available from: http://dx.doi.org/10.1093/schbul/sbu078

14. Golland P, Fischl B. Permutation tests for classification: towards statistical significance in image-based studies. Inf Process Med Imaging [Internet]. 2003 Jul;18:330–41. Available from: http://dx.doi.org/10.1007/978-3-540-45087-0_28

15. Koutsouleris N, Wobrock T, Guse B, Langguth B, Landgrebe M, Eichhammer P, et al. Predicting Response to Repetitive Transcranial Magnetic Stimulation in Patients With Schizophrenia Using Structural Magnetic Resonance Imaging: A Multisite Machine Learning Analysis [Internet]. Vol. 44, Schizophrenia Bulletin. 2018. p. 1021–34. Available from: http://dx.doi.org/10.1093/schbul/sbx114

16. Deegener G. Multiphasic Sex Inventory (MSI). Göttingen, Germany: Hogrefe Verlag; 1996. 308 p.

17. Benjamini Y, Hochberg Y. Controlling the false discovery rate: a practical and powerful approach to multiple testing. J R Stat Soc. 1995;

18. Zhou Y, Shi L, Cui X, Wang S, Luo X. Functional Connectivity of the Caudal Anterior Cingulate Cortex Is Decreased in Autism. PLoS One [Internet]. 2016 Mar 17;11(3):e0151879. Available from: http://dx.doi.org/10.1371/journal.pone.0151879

19. Kelly AMC, Di Martino A, Uddin LQ, Shehzad Z, Gee DG, Reiss PT, et al. Development of anterior cingulate functional connectivity from late childhood to early adulthood. Cereb Cortex [Internet]. 2009 Mar;19(3):640–57. Available from: http://dx.doi.org/10.1093/cercor/bhn117

20. Motomura Y, Kitamura S, Nakazaki K, Oba K, Katsunuma R, Terasawa Y, et al. Recovery from Unrecognized Sleep Loss Accumulated in Daily Life Improved Mood Regulation via Prefrontal Suppression of Amygdala Activity. Front Neurol [Internet]. 2017 Jun 30;8:306. Available from: http://dx.doi.org/10.3389/fneur.2017.00306
